# Associations between insulin-like growth factors and their binding proteins and other prognostic indicators in breast cancer.

**DOI:** 10.1038/bjc.1996.523

**Published:** 1996-10

**Authors:** H. Yu, M. A. Levesque, M. J. Khosravi, A. Papanastasiou-Diamandi, G. M. Clark, E. P. Diamandis

**Affiliations:** Department of Pathology and Laboratory Medicine, Mount Sinai Hospital, Toronto, Ontario, Canada.

## Abstract

Recent studies have suggested that insulin-like growth factors (IGFs) and insulin-like growth factor binding proteins (IGFBPs) may be implicated in the development and progression of breast cancer. Prostate-specific antigen (PSA), a serine protease, may play a role in the regulation of IGFs' function through cleavage of IGFBP-3, resulting in release of active IGFs from IGFBP-3. As IGFs, IGFBPs and PSA are all present in breast cancer, possible associations among these proteins were speculated. In this study, we have measured PSA, IGF-I, IGF-II, IGFBP-1 and IGFBP-3 in tumour tissue cytosols from 200 women with primary breast cancer, and have examined relationships between IGFs or IGFBPs and PSA along with other markers, including p53 protein, steroid hormone receptors (oestrogen and progesterone), cathepsin-D, epidermal growth factor receptor, Her-2/neu protein, S-phase fraction and DNA ploidy. Correlations or associations between PSA and IGF-I, IGF-II, IGFBP-1 or IGFBP-3 were not observed. IGF-II was positively correlated with both IGFBP-3 and IGFBP-1. IGF-I was not associated with either of the two binding proteins, nor with IGF-II. Both IGF-II and IGFBP-3 were inversely associated with the oestrogen receptor, and IGFBP-3 was also positively associated with S-phase fraction. Our finding of IGF-II and IGFBP-3 in association with unfavourable prognostic indicators of breast cancer suggests that IGFs may be involved in the progression of breast cancer.


British Journal of Cancer (1996) 74, 1242-1247
© 1996 Stockton Press All rights reserved 0007-0920/96 $12.00

### Associations between insulin-like growth factors and their binding proteins and other prognostic indicators in breast cancer

H Yu,[1,2] MA Levesque,[1,2] MJ Khosravi,[2,3] A Papanastasiou-Diamandi,[3] GM Clark[4] and EP Diamandis[1,2]

[1]*Department of Pathology and Laboratory Medicine, Mount Sinai Hospital, 600 University Avenue, Toronto, Ontario, Canada M5G 1X5;* [2]*Department of Clinical Biochemistry, University of Toronto, 100 College Street, Toronto, Ontario, Canada M5G 1L5;* [3]*Diagnostic Systems Laboratories (Canada), 600 University Avenue, Toronto, Ontario, Canada M5G 1X5;* [4]*Department of Medicine, Division of Medical Oncology, University of Texas Health Science Center at San Antonio, 7703 Floyd Curl Drive, San Antonio, Texas 78284-7884, USA.*

**Summary** Recent studies have suggested that insulin-like growth factors (IGFs) and insulin-like growth factor binding proteins (IGFBPs) may be implicated in the development and progression of breast cancer. Prostate-specific antigen (PSA), a serine protease, may play a role in the regulation of IGFs' function through cleavage of IGFBP-3, resulting in release of active IGFs from IGFBP-3. As IGFs, IGFBPs and PSA are all present in breast cancer, possible associations among these proteins were speculated. In this study, we have measured PSA, IGF-I, IGF-II, IGFBP-1 and IGFBP-3 in tumour tissue cytosols from 200 women with primary breast cancer, and have examined relationships between IGFs or IGFBPs and PSA along with other markers, including p53 protein, steroid hormone receptors (oestrogen and progesterone), cathepsin-D, epidermal growth factor receptor, Her-2/neu protein, S-phase fraction and DNA ploidy. Correlations or associations between PSA and IGF-I, IGF-II, IGFBP-1 or IGFBP-3 were not observed. IGF-II was positively correlated with both IGFBP-3 and IGFBP-1. IGF-I was not associated with either of the two binding proteins, nor with IGF-II. Both IGF-II and IGFBP-3 were inversely associated with the oestrogen receptor, and IGFBP-3 was also positively associated with S-phase fraction. Our finding of IGF-II and IGFBP-3 in association with unfavourable prognostic indicators of breast cancer suggests that IGFs may be involved in the progression of breast cancer.

**Keywords:** breast cancer; prognostic marker; growth factor; insulin-like growth factor binding protein; prostate specific antigen

Insulin-like growth factors (IGFs), including IGF-I and IGF-II, belong to a family of peptide hormones involved in the regulation of normal cell growth and differentiation. IGFs exert their action through binding to two types of specific receptors, one of which (IGF-I receptor) is a transmembrane protein with tyrosine kinase activity (Krywicki and Yee, 1992; Daughaday and Rotwein, 1989). The binding of IGFs to their receptors is modulated by a group of soluble proteins called insulin-like growth factor binding proteins (IGFBPs). Six distinct IGFBPs have been described so far (IGFBP-1 to IGFBP-6). Since only the free IGFs are able to interact with their cell surface receptors, IGFBPs regulate the bioavailability and bioactivity of IGFs through binding to them (Figueroa and Yee, 1992). IGFBP-3 may also bind to its own receptor on the cell membrane and functions independently from the IGFs (Oh *et al.*, 1993; Valentinis *et al.*, 1995).

Loss of functional IGF-I receptor was shown to be associated with slow cell growth and prolonged cell cycle. Moreover, the receptor was found to be involved in cell transformation and apoptosis (LeRoith *et al.*, 1995; Baserga, 1995). Therefore, it was speculated that IGFs may play a role in the development and progression of cancer. Experimental studies have shown that IGFs could act as mitogens and promote the growth of breast tumour cells (Arteaga *et al.*, 1992) and that IGFBPs regulate the impact of IGFs on tumour cells (Figueroa and Yee, 1992). The presence of IGFs and their binding proteins in breast cancer cells has been well characterised and their relationships have been studied in both cultured breast cancer cell lines as well as breast cancer tissues (Cullen *et al.*, 1992; Yee *et al.*, 1991; Sheikh *et al.*, 1992; McGuire *et al.*, 1992; Pekonen *et al.*, 1992; Paik 1992).

Responses of breast cancer cells to tamoxifen treatment were found to be associated with changes in IGFs and IGFBPs; it was shown IGF-I levels decreased and IGFBP-3 levels increased in the serum of breast cancer patients after administration of tamoxifen (Pollak *et al.*, 1990; Lonning *et al.*, 1992). In animal experiments, tamoxifen was shown to suppress the expression of the IGF-I gene (Huynh *et al.*, 1993). The association between tamoxifen treatment and IGFs or IGFBPs suggests that the production of IGFs or IGFBPs may be regulated by steroid hormones (Pratt and Pollak, 1993, 1994; Owens *et al.*, 1993; Winston *et al.*, 1994; Manni *et al.*, 1994). The presence of the oestrogen receptor (ER) or the progesterone receptor (PR) has also been found to be associated with levels of both IGFs and IGFBPs in breast cancer cell lines and tissues (Figueroa *et al.*, 1993; Yee *et al.*, 1994).

Prostate-specific antigen (PSA) is a glycoprotein and serine protease with chymotrypsin-like enzymatic activity. PSA has been found to be able to cleave IGFBP-3 (Cohen *et al.*, 1992), suggesting that PSA may regulate the function of IGFs through the digestion of IGFBP-3 (Cohen *et al.*, 1994; Kanety *et al.*, 1993). Correlations between PSA and some IGFBPs have been observed in the serum of prostate cancer patients (Kanety *et al.*, 1993). PSA, currently used as a biochemical marker for diagnosis and management of patients with prostate cancer, was initially thought to be produced exclusively by the prostate, but recently it has been found in breast cancer tissue (Diamandis *et al.*, 1994). The presence of PSA in breast cancer was associated with steroid hormone receptors, and the breast cancer cell lines could produce PSA after the cells were stimulated with androgens or progestins (Yu *et al.*, 1994). Based on these observations, we speculate that associations may exist among IGFBPs, IGFs and PSA in breast cancer. In order to investigate this

Correspondence: EP Diamandis, Department of Pathology and Laboratory Medicine, Mount Sinai Hospital, 600 University Ave., Toronto, Ontario M5G 1X5, Canada
Received 29 Janurary 1996; revised 16 April 1996; accepted 1 May 1996

possibility, we have analysed 200 breast cancer tissues with immunoassays for IGF-I, IGF-II, IGFBP-1, IGFBP-3 and PSA. We have also examined these markers in association with other prognostic indicators in breast cancer, including p53 protein, steroid hormone receptors (ER and PR), cathepsin-D (CATD), epidermal growth factor receptor (EGFR), Her-2/neu protein (HER-2), S-phase fraction (SPF) and DNA ploidy. The findings of the study are reported in this paper.

### Materials and methods

### *Breast cancer specimens and cytosol extraction*

Tumour tissue specimens from 200 patients with primary breast cancer were selected from the tissue bank in the University of Texas Health Science Center at San Antonio. The specimens, stored at $-80°C$ until the extraction of tissue cytosols for this project, were banked after analysis of ER, PR, SPF and DNA ploidy. Also analysed were other biochemical markers including EGFR, CATD and HER-2. Clinical and follow-up information of these patients was not available.

The cytosolic extracts were prepared in a lysis buffer after about 200 mg of frozen tissue specimens were pulverised into a fine powder. The lysis buffer, pH 8.0, contains per litre 50 mmol Tris, 150 mmol sodium chloride, 5 mmol EDTA, 10 g Nonidet NP-40, 100 mg phenylmethylsulphonyl fluoride (PMSF) and 1 mg each of aprotinin and leupeptin. The tissue powder was lysed in 1 ml of the lysis buffer for 30 min on ice. The lysates were then centrifuged at 4°C at 15 000 ***g*** for 30 min. The supernatants were assayed for PSA, p53, IGF-I, IGF-II, IGFBP-1, IGFBP-3 and total protein.

### *Measurement of biochemical markers*

IGF-I, IGF-II, IGFBP-1 and IGFBP-3 were measured with immunoassay kits commercially available from Diagnostic Systems Laboratories (DSL, Webster, TX, USA). The assays were based on a non-competitive, sandwich-type principle involving a solid-phase capture antibody and a soluble $^{125}I$- or horseradish peroxidase (HRP)-labelled detection antibody. All assays were performed according to the manufacturer's protocols. A brief description of the assays is as follows: The DSL IGF-I kit is a one-step enzyme-linked immunosorbent assay (ELISA), involving anti-IGF-I antibody-coated microtitration wells and a monoclonal anti-IGF-I detection antibody labelled with HRP. The IGF-I kit has a standard range of $0.1-6\ \mu g\ l^{-1}$ and a precision of $<10\%$ coefficient of variation (CV).

The DSL IGF-II kit is a two-step immunoradiometric assay (IRMA), involving anti-IGF-II antibody-coated tubes and a radioiodinated monoclonal detection antibody. The IRMA assay has a standard range between 3 and $50\ \mu g\ l^{-1}$. The assay's precision is $<10\%$ CV. Both IGF-I and IGF-II assays require an acid–ethanol extraction step which is designed to dissociate and remove IGFBPs from the samples before analysis. The extraction procedures include sequential mixture of $50\ \mu l$ sample with $450\ \mu l$ extraction solution at room temperature for 30 min, centrifugation of the mixture at 10 000 r.p.m. for 3 min, mixture of the supernatant at 1:1 ratio with neutralisation solution, and assay of the final mixture diluted (1:5) in an assay buffer (a final dilution of sample 1:100).

The DSL IGFBP-1 and IGFBP-3 kits are also based on ELISA principles. The assays were performed in microtitration wells coated with a highly specific anti-IGFBP-1 or IGFBP-3 antibody. The IGFBP-3 ELISA kit uses a polyclonal detection antibody, while a monoclonal antibody is used in the IGFBP-1 kit. Both assays are based on a two-step assay principle and incorporate HRP as label. The calibrator ranges are between 0.25 and $25\ \mu g\ l^{-1}$ for the IGFBP-1 kit and between 2 and $100\ \mu g\ l^{-1}$ for the IGFBP-3 kit. The precision is $<10\%$ CV for both assays.

The methods used for the measurement of ER, PR, EGFR, CATD, HER-2, SPF, DNA ploidy, p53, PSA and total protein have been described previously (Levesque *et al.*, 1995). Briefly, ER and PR were measured by the dextran-coated charcoal method. EGFR was determined by a radiolabelled ligand-binding assay. An EIA kit from Triton Diagnostics was used to measure CATD, and HER-2 was measured by the Western blotting technique. DNA flow cytometry was used to determine DNA content and S-phase fraction, which was defined as the percentage of cells in S phase among the diploid populations. Based on the previous study, less than 6.7% of S-phase fraction was considered a cut-off for favourable prognosis. PSA and p53 were measured by two in-house sandwich-type time-resolved immunofluorometric assays. For the samples which were used for the measurements of ER, PR, EGFR, CATD and HER-2, the total protein was determined by the Lowry method, and for those used for PSA and p53 measurement, the protein was measured by a bicinchoninic acid-based commercial kit from Pierce.

The measurements of ER, PR, EGFR, CATD, HER-2, SPF and DNA ploidy were performed in San Antonio when the specimens were collected, whereas PSA, p53, IGFs and IGFBPs were determined later in Toronto when the present study was undertaken.

### *Statistical analysis*

As most of the data generated by the immunoassays or other analytical methods do not follow Gaussian distributions, the numerical values were analysed non-parametrically. The Spearman correlation coefficients were calculated for assessing the correlation between any two of the markers, and the Wilcoxon rank sum test was employed to compare the median concentrations between groups. The numerical data were also classified into categories, and were analysed using chi-square or Fisher's exact tests. The cut-off values for the categorical group were either based on the percentile boundary (median) or were established elsewhere (Levesque *et al.*, 1995). For IGFs and IGFBPs, the values were grouped into high and low categories based on the medians. PSA and p53 were classified into positive and negative groups using cut-off levels of $0.03\ ng\ mg^{-1}$ and $5\ units\ g^{-1}$ respectively. Positive and negative classifications were also used for ER ($3\ fmol\ mg^{-1}$), PR ($5\ fmol\ mg^{-1}$), EGFR ($10\ fmol\ mg^{-1}$) and CATD ($51\ pmol\ mg^{-1}$). HER-2 was categorised into high and low groups following the method described by Tandon *et al.* (1989).

### Results

Because of the lack of enough sample volume in some specimens, of the 200 samples, 135 were measured for IGF-I and IGF-II, 160 were measured for IGFBP-1, and 169 were measured for IGFBP-3. The frequency distributions of the values of IGFs and IGFBPs are shown in Figures 1–4. The numerical distributions of the four markers are demonstrated in Table I. For IGF-I, the lowest value was $0.9\ ng\ mg^{-1}$ and the highest $52\ ng\ mg^{-1}$. The median and mean were close, 7 and $8\ ng\ mg^{-1}$ respectively. In comparison to IGF-I, the IGF-II levels were much higher, ranging from 4.2 to $72\ ng\ mg^{-1}$ with an identical median and mean value of $36\ ng\ mg^{-1}$. With regard to the two binding proteins, IGFBP-3 concentration was much higher than IGFBP-1. The values ranged from 0.02 to $6.6\ ng\ mg^{-1}$ for IGFBP-1 and from 0.2 to $369\ ng\ mg^{-1}$ for IGFBP-3. The median values were all lower than the means for the two markers, suggesting a positive skew of the data from the normal distribution (Table I).

The correlations between any two of these markers are shown in Table II. Among the two IGFs and two IGFBPs, the levels of IGFBP-3 were positively correlated with IGFBP-1 ($r=0.20$, $P=0.02$) and IGF-II ($r=0.38$, $P<0.01$), but not with IGF-I ($r=0.10$, $P=0.27$). IGFBP-1 was not correlated

**Figure 1** Frequency distribution of IGF-I.

**Figure 2** Frequency distribution of IGF-II.

**Figure 3** Frequency distribution of IGFBP-1.

**Figure 4** Frequency distribution of IGFBP-3.

with either of the IGFs ($r=-0.08$, $P=0.36$ for IGF-I; $r=0.05$, $P=0.54$ for IGF-II). The two IGFs did not correlate to each other ($r=0.08$, $P=0.35$).

PSA levels were not correlated with any of the four markers, IGF-I ($r=-0.03$, $P=0.72$), IGF-II ($r=-0.12$, $P=0.17$), IGFBP-1 ($r=-0.01$, $P=0.85$) or IGFBP-3 ($r=-0.03$, $P=0.72$). An inverse correlation was seen between ER and IGF-II ($r=-0.19$, $P=0.03$) or IGFBP-3 ($r=-0.31$, $P<0.01$), and a similar correlation was also suggested between PR and IGFBP-3 ($r=-0.16$, $P=0.04$). IGF-II was positively correlated with p53 ($r=0.27$, $P<0.01$), and was negatively correlated with CATD ($r=-0.19$, $P=0.02$). IGFBP-3 was positively correlated with EGFR ($r=0.17$, $P=0.03$) and SPF ($r=0.27$, $P<0.01$). Most of the correlations were relatively weak; their correlation coefficients were less than 0.3.

Tables III and IV demonstrate the associations between any two of the markers studied when their values were classified into two groups. IGFs and IGFBPs were grouped into high and low categories based on their median values. Other markers were classified either into positive *vs* negative groups or into high and low categories based on specific cut-off levels. The results of this analysis were quite similar to those of the correlation analysis for the relationships between

**Table I** Distribution of IGF-I, IGF-II, IGFBP-1 and IGFBP-3 levels in breast cancer cytosols

| | Percentile (ng $mg^{-1}$)[a] | | | | | |
|---|---|---|---|---|---|---|
| *Marker* | *0%* | *25%* | *50%* | *75%* | *100%* | *Mean* |
| IGF-I | 0.9 | 5.3 | 7.0 | 9.5 | 52 | 8.0 |
| IGF-II | 4.2 | 29 | 36 | 41 | 72 | 36 |
| IGFBP-1 | 0.02 | 0.15 | 0.32 | 0.62 | 6.6 | 0.51 |
| IGFBP-3 | 0.2 | 44 | 63 | 95 | 369 | 81 |

[a]Values are in ng of marker per mg of total protein in the cytosolic extract.

**Table II** Spearman correlation analysis between the prognostic markers

| *Marker* | *IGF-I* | *IGF-II* | *IGFBP-1* | *IGFBP-3* |
|---|---|---|---|---|
| PSA | | | | |
| *r*[a] | −0.03 | −0.12 | −0.01 | −0.03 |
| *P*-value | 0.72 | 0.17 | 0.85 | 0.72 |
| ER | | | | |
| *r* | −0.15 | −0.19 | 0.08 | −0.31 |
| *P*-value | 0.09 | 0.03 | 0.30 | <0.01 |
| PR | | | | |
| *r* | 0.01 | −0.10 | 0.11 | −0.16 |
| *P*-value | 0.89 | 0.23 | 0.17 | 0.04 |
| p53 | | | | |
| *r* | −0.02 | 0.27 | −0.08 | 0.06 |
| *P*-value | 0.84 | <0.01 | 0.33 | 0.43 |
| CATD[b] | | | | |
| *r* | −0.16 | −0.19 | 0.15 | 0.01 |
| *P*-value | 0.06 | 0.02 | 0.06 | 0.91 |
| EGFR[c] | | | | |
| *r* | −0.05 | −0.03 | −0.01 | 0.17 |
| *P*-value | 0.54 | 0.73 | 0.90 | 0.03 |
| SPF[d] | | | | |
| *r* | 0.004 | 0.14 | −0.03 | 0.27 |
| *P*-value | 0.96 | 0.10 | 0.72 | <0.01 |
| IGFBP-3 | | | | |
| *r* | 0.10 | 0.38 | 0.20 | |
| *P*-value | 0.27 | <0.01 | 0.02 | |
| IGFBP-1 | | | | |
| *r* | −0.08 | 0.05 | | |
| *P*-value | 0.36 | 0.54 | | |
| IGF-II | | | | |
| *r* | 0.08 | | | |
| *P*-value | 0.35 | | | |

[a]*r*, Spearman correlation coefficient; [b]CATD, cathepsin D; [c]EGFR, epidermal growth factor receptor [d]SPF, S-phase fraction.

Table III Associations between prognostic markers and IGFs

| Marker (%) | Total | IGF-I High[a] | | IGF-I Low[b] | IGF-II High[a] | | IGF-II Low[b] |
|---|---|---|---|---|---|---|---|
| PSA (+) | 25.2 | 27.1 | | 23.1 | 27.1 | | 23.1 |
| P-value | | | 0.59 | | | 0.59 | |
| ER (+) | 83.7 | 82.9 | | 84.6 | 77.1 | | 90.8 |
| P-value | | | 0.78 | | | 0.03 | |
| PR (+) | 61.5 | 64.3 | | 58.5 | 54.3 | | 69.2 |
| P-value | | | 0.49 | | | 0.08 | |
| p53 (+) | 23.7 | 22.9 | | 24.6 | 25.7 | | 21.5 |
| P-value | | | 0.81 | | | 0.57 | |
| CATD (+) | 31.9 | 22.9 | | 41.5 | 24.3 | | 40.0 |
| P-value | | | 0.02 | | | 0.05 | |
| EGFR (+) | 24.4 | 24.3 | | 24.6 | 25.7 | | 23.1 |
| P-value | | | 0.96 | | | 0.72 | |
| HER2 (+) | 22.2 | 20.0 | | 24.6 | 21.4 | | 23.1 |
| P-value | | | 0.52 | | | 0.82 | |
| SPF≥6.7% | 37.0 | 35.7 | | 38.5 | 40.0 | | 33.9 |
| P-value | | | 0.74 | | | 0.46 | |
| Diploid | 45.9 | 50.0 | | 41.5 | 40.0 | | 52.3 |
| P-value | | | 0.32 | | | 0.15 | |
| IGFBP-1 (+) | 48.5 | 45.7 | | 51.7 | 47.8 | | 49.2 |
| P-value | | | 0.50 | | | 0.87 | |
| IGFBP-3 (+) | 52.6 | 56.5 | | 48.4 | 68.6 | | 34.9 |
| P-value | | | 0.35 | | | <0.01 | |

[a]High: IGF values higher than or equal to the median level. [b]Low: IGF values lower than the median level.

Table IV Associations between prognostic markers and IGFBPs

| Marker (%) | Total | IGFBP-I High[a] | | IGFBP-I Low[b] | IGFBP-II High[a] | | IGFBP-II Low[b] |
|---|---|---|---|---|---|---|---|
| PSA (+) | 26.3 | 24.1 | | 28.4 | 26.7 | | 30.1 |
| P-value | | | 0.53 | | | 0.63 | |
| ER (+) | 85.6 | 81.0 | | 90.1 | 77.9 | | 92.8 |
| P-value | | | 0.10 | | | 0.01 | |
| PR (+) | 61.9 | 63.3 | | 60.5 | 54.7 | | 68.7 |
| P-value | | | 0.72 | | | 0.06 | |
| p53 (+) | 21.9 | 20.3 | | 23.5 | 26.7 | | 18.1 |
| P-value | | | 0.62 | | | 0.18 | |
| CATD (+) | 34.4 | 40.5 | | 28.4 | 36.1 | | 32.5 |
| P-value | | | 0.11 | | | 0.63 | |
| EGFR (+) | 23.1 | 21.5 | | 24.7 | 25.6 | | 22.9 |
| P-value | | | 0.63 | | | 0.68 | |
| HER2 (+) | 20.0 | 21.5 | | 18.5 | 22.1 | | 16.9 |
| P-value | | | 0.64 | | | 0.39 | |
| SPF≥6.7% | 36.3 | 43.0 | | 29.6 | 47.7 | | 24.1 |
| P-value | | | 0.08 | | | <0.01 | |
| Diploid | 47.5 | 41.8 | | 53.1 | 39.5 | | 51.8 |
| P-value | | | 0.15 | | | 0.11 | |

[a]High: IGF values higher than or equal to the median level. [b]Low: IGF values lower than the median level.

IGFs and IGFBPs or between IGFs or IGFBPs and PSA, ER, CATD or SPF. However, unlike the results of the correlation analysis, a positive association between p53 and IGF-II was not found, nor was an association of IGFBP-3 with EGFR observed.

IGF-II was positively associated with IGFBP-3 (69% of high-level IGFBP-3 in high IGF-I group *vs* 35% in low IGF-II group, $P<0.01$). PSA positivity did not show any association with either of the IGFs (27% in high and 23% in low IGFs, $P=0.59$) or either of the IGFBPs (24% or 27% in high and 28% or 30% in low IGFBPs, $P=0.53$ or 0.63). ER status was inversely associated with IGF-II (77% of ER positivity in high IGF-II group *vs* 91% in low IGF-II group, $P=0.03$) or with IGFBP-3 (78% and 93% of ER positivity in high and low IGFBP-3 respectively, $P=0.01$). A similar but weak association between PR and IGFBP-3 was also observed (55% *vs* 69%, $P=0.06$). An inverse association was suggested between CATD and both IGFs (23% or 24% of high-level CATD in high IGFs *vs* 42% or 40% in low IGFs, $P=0.02$ or 0.05). SPF was positively associated with IGFBP-3 (48% of SPF in high *vs* 24% in low IGFBP-3, $P<0.01$).

DNA ploidy, p53 and HER-2 protein did not show any association with either IGFs or IGFBPs. The results of the Wilcoxon rank sum test which compared the median values of IGFs or IGFBPs between two categories of another marker, such as PSA-positive *vs* PSA-negative groups, were similar to those of chi-square tests (data not shown).

### Discussion

Given the fact that IGFs bind to other proteins in serum or cells, an extraction step which separates the IGFs from their binding proteins has been included in the procedure of IGF measurement. The acid–ethanol method is used for the extraction. The efficiency of the extraction has been evaluated by comparing the IGF values in 20 serum samples extracted by the acid–ethanol method with those extracted by acid gel filtration chromatography, a method regarded as a standard for extraction. IGF levels in the samples extracted by the two methods were highly correlated to each other ($r=0.98$) (Khosravi *et al.*, 1996).

Among the two IGFs and two IGFBPs, IGF-II and IGFBP-3 were shown to have a strong positive correlation; the Spearman correlation coefficient was 0.38 ($P<0.01$). IGFBP-1 was also significantly correlated with IGFBP-3 ($r=0.20$; $P=0.02$). No correlation was found between the two IGFs. IGF-I did not correlate to either of the IGFBPs. A positive correlation between IGF-II and IGFBP-3 was also suggested in a study which found that the mRNA levels of IGF-II and IGFBP-3 declined after the reduction of oestrogen level, and were elevated again when the oestrogen level increased (Manni *et al.*, 1994).

PSA digestion of IGFBP-3 has been observed previously in seminal plasma (Cohen *et al.*, 1992). Experimental study showed that this digestion could result in recovery of the mitogenic effect of IGFs on prostatic cells, which was believed to be suppressed by IGFBP-3 (Cohen *et al.*, 1994). An inverse correlation between IGFBP-3 and PSA was also observed in the serum of prostate cancer patients (Kanety *et al.*, 1993). More recently, it was found that IGF-I was able to activate the androgen receptor directly (Culig *et al.*, 1994). Androgen, the legitimate ligand of androgen receptor, is known to up-regulate the transcription of PSA mRNA (Young *et al.*, 1991). PSA was initially thought to be produced specifically by the prostate, but our recent studies demonstrated the presence of PSA in breast cancer tissue (Diamandis *et al.*, 1994). If PSA was a protease of IGFBP-3 in breast tissue, a correlation or association between PSA and IGFBP-3 might be expected. However, such a relationship was not observed in this study. This may suggest that the cleavage of IGFBP-3 by PSA does not occur in breast cancer or that PSA in the breast is not enzymatically active.

Other explanations for our finding of no association between PSA and IGFBP-3 in breast cancer may also exist. One of the possibilities could be the method we used to measure IGFBP-3. It is known that IGFBP-3 is present in the circulation or tissues in two major molecular forms, intact IGFBP-3 and IGFBP-3 fragments (Gargosky *et al.*, 1992). The fragments are the products of proteolytic cleavage by the proteases of IGFBP-3. The total amount of IGFBP-3 (intact plus fragments) may remain the same after the proteolytic process, while the ratio of the intact IGFBP-3 and its fragments undergoes substantial changes. Our method for IGFBP-3 is a sandwich-type immunoassay which quantifies both intact IGFBP-3 and IGFBP-3 fragments. The method used by others for prostate cancer study (Kanety et al., 1993) was a ligand-binding assay, which detected only the intact IGFBP-3, as the binding affinity of IGFBP-3 to its ligand IGFs is dependent on the complete structure of IGFBP-3. Fragmented IGFBP-3 loses its ligand-binding ability. Therefore, the levels of IGFBP-3 measured by the ligand-binding method should be lower than those measured by our method, and the two measurements may suggest different status of the protein and its relationships with other substances. It would provide additional information if the two types of methods could have been compared in this study.

Since both PSA and IGFs along with their binding proteins are associated with steroid hormone regulation, it was speculated that associations between PSA and IGFBPs or IGFs might be present only in the subgroups of patients who were positive for the steroid hormone receptors. Based on this speculation, we further examined the association between PSA and IGFBPs or IGFs within a subgroup of tumours which were either ER positive or negative. However, the results remained the same as those without the adjustment for steroid hormone receptor.

The presence of ER or PR in breast cancer is known to relate to a good prognosis of breast cancer patients, whereas high levels of p53 protein, CATD, EGFR or HER-2 protein are thought to be associated with unfavourable outcome of the disease (McGuire et al., 1990; McGuire and Clark, 1992). High percentage of SPF or aneuploid DNA is also believed to be indicative of poor prognosis. With regard to PSA, it was recently found by our group that the presence of PSA could suggest a favourable outcome of breast cancer (Yu et al., 1995). Combining all the findings in the study, we observed that high levels of IGF-II and IGFBP-3 tended to be related to unfavourable prognostic indicators of breast cancer. It included an inverse correlation as well as association between ER and IGF-II or IGFBP-3, and a positive correlation and association between IGFBP-3 and SPF. There was also a positive correlation but not a positive association between IGF-II and p53 and between IGFBP-3 and EGFR.

Under the overall trend of IGFs and IGFBPs in association with poor prognostic markers of breast cancer, CATD was the only exception. CATD was shown in the study to have an inverse correlation as well as association with both IGF-II and IGF-I. However, many studies have demonstrated that CATD is an indicator for poor prognosis. We were unable to find a reasonable explanation for this observation.

Our finding of IGF-II and IGFBP-3 in association with poor prognostic markers of breast cancer is consistent with the findings demonstrated by others. An inverse relationship between ER and IGFBP-3 has been reported in a number of studies (Yee et al., 1991; Manni et al., 1994; Figueroa et al., 1993; Clemmons et al., 1990). Cell culture studies demonstrated that IGFs as mitogens could facilitate the growth of tumour cells and increase the resistance of cells to apoptosis (Baserga, 1995). IGF levels were reduced in patients who responded well to tamoxifen treatment (Pollak et al., 1992).

Since IGFBP-1 and -3 are not the only binding proteins present in breast cancer cells, the real relationships between IGFs and IGFBPs and between IGF axis and other proteins will not be fully understood until all other binding proteins (i.e. IGFBP-2, -4, -5 and -6) are under investigation. Further studies that investigate both IGFs and all the binding proteins are needed.

Studies on tumour tissue cytosols inherit a potential limitation, which is the homogeneity of the tissue specimen. Tumour tissues from different patients may contain different amounts of tumour cells and other tissues, such as connective tissues and blood vessels. This variation may in turn result in variations in our measurement of various protein markers. In order to control for this variation, we measured the tumour cell content in each tissue specimen by cytometry. About 72% of tissue samples contained more than 50% tumour cells, and 26% samples had tumour cell content between 20% and 50%. Only 2% of specimens had tumour cell content between 10% and 20%. The variation of tumour cell volume among these samples is relatively limited. A substantial impact of tissue homogeneity on our observations should not be expected.

It remains unknown if the measurement of IGFs and IGFBPs in the serum or tissue extracts of breast cancer patients will have any clinical implication. A recent study examined the levels of IGFBPs in tumour cytosols in association with the survival of patients with node-negative breast cancer. It was found that patients with low levels of IGFBP-4 in their tumour had longer disease-free survival, and this association was seen only in patients with large tumours. Other IGFBPs did not show any relationship with survival (Yee et al., 1994). Unfortunately, we could not examine the relationship between IGFs or IGFBPs and patient survival in this study since the follow-up information of these patients was not available.

In summary, IGF-I, IGF-II, IGFBP-1 and IGFBP-3 levels were measured in breast cancer tissue with immunoassays. A positive correlation was seen between IGF-II and IGFBP-3. IGFBP-1 was also weakly associated with IGFBP-3, but was not associated with either of the IGFs. IGF-I was associated with neither of the binding proteins, nor with IGF-II. High levels of IGF-II and IGFBP-3 tended to be related to unfavourable prognostic markers of the cancer, such as high-level SFP or ER negative status. Correlations or associations between PSA and IGFBPs or IGFs were not observed. As suggested in other studies, IGF-II and IGFBP-3 seem to be involved in the development and/or progression of breast cancer. Studies to clarify further their role in breast cancer are needed.

### Abbreviations

IGFs, insulin-like growth factors; IGFBPs, insulin-like growth factor binding proteins; IGF-I, insulin-like growth factor I; IGF-II, insulin-like growth factor II; IGFBP-1, insulin-like growth factor binding protein 1; IGFBP-3, insulin-like growth factor binding protein 3; PSA, prostate-specific antigen; ER, oestrogen receptor; PR, progesterone receptor; CATD, cathepsin D; EGFR, epidermal growth factor receptor; HER-2, Her-2/neu protein; SPF, S-phase fraction; HRP, horseradish peroxidase; ELISA, enzyme-linked immunosorbent assay; IRMA, immunoradiometric assay.

### Acknowledgement

This work was supported by NCI Grant CA58183.

### References

ARTEAGA CL. (1992). Interference of the IGF system as a strategy to inhibit breast cancer growth. *Breast Cancer Res. Treat.*, **22,** 101–106.

BASERGA R. (1995). The insulin-like growth factor I receptor: a key to tumor growth? *Cancer Res.*, **55,** 249–252.

CLEMMONS DR, CAMACHO-HUBNER C, CORONADO E AND OSBORNE CK. (1990). Insulin-like growth factor binding protein secretion by breast cancer cell lines: Correlation with estrogen receptor status. *Endocrinology*, **127,** 2679–2686.

COHEN P, GRAVES HCB, PEEHL DM, KAMAREI M, GIUDICE LC, AND ROSENFELD RG. (1992). Prostate-specific antigen (PSA) is an insulin-like growth factor binding protein-3 protease found in seminal plasma. *J. Clin. Endocrinol. Metab.*, **75,** 1046–1053.

COHEN P, PEEHL DM, GRAVES HCB AND ROSENFELD RG. (1994). Biological effects of prostate specific antigen as an insulin-like growth factor binding protein-3 protease. *J. Endocrinol.*, **142,** 407–415.

CULIG Z, HOBISCH A, CRONAUER MV, RADMAYR C, TRAPMAN J, HITTMAIR A, BARTSCH G AND KLOCKER H. (1994). Androgen receptor activation in prostatic tumor cell lines by insulin-like growth factor-I, keratinocyte growth factor, and epidermal growth factor. *Cancer Res.*, **54**, 5474–5478.

CULLEN KJ, ALLISON A, MARTIRE I, ELLIS M AND SINGER C. (1992). Insulin-like growth factor expression in breast cancer epithelium and stroma. *Breast Cancer Res. Treat.*, **22**, 21–29.

DAUGHADAY WH AND ROTWEIN P. (1989). Insulin-like growth factors I and II. Peptide, messenger ribonucleic acid and gene structure, serum, and tissue concentrations. *Endocr. Rev.*, **10**, 68–91.

DIAMANDIS EP, YU H AND SUTHERLAND DJA. (1994). Detection of prostate-specific antigen immunoreactivity in breast tumors. *Breast Cancer Res. Treat.*, **32**, 301–310.

FIGUEROA JA AND YEE D. (1992). The insulin-like growth factor binding proteins (IGFBPs) in human breast cancer. *Breast Cancer Res. Treat.*, **22**, 81–90.

FIGUEROA JA, JACKSON JG, MCGUIRE WL, KRYWICKI RF AND YEE D. (1993). Expression of insulin-like growth factor binding proteins in human breast cancer correlations with estrogen receptor status. *J. Cell Biochem.*, **52**, 196–205.

GARGOSKY SE, PHAM HM, WILSON KF, LIU F, GIUDICE LC AND ROSENFELD RG. (1992). Measurement and characterization of insulin-like growth factor binding protein-3 in human biological fluids: discrepancies between radioimmunoassay and ligand blotting. *Endocrinology*, **131**, 3051–3060.

HUYNH HT, TETENES E, WALLACE L AND POLLAK M. (1993). *In vivo* inhibition of insulin-like growth factor I gene expression by tamoxifen. *Cancer Res.*, **53**, 1727–1730.

KANETY H, MADJAR Y, DAGAN Y, LEVI J, PAPA MZ, PARIENTE C, GOLDWASSER B AND KARASIK A. (1993). Serum insulin-like growth factor-binding protein-2 (IGFBP-2) is increased and IGFBP-3 is decreased in patients with prostate cancer: correlation with serum prostate-specific antigen. *J. Clin. Endocrinol. Metab.*, **77**, 229–233.

KHOSRAVI MJ, DIAMANDI A, MISTRY J AND LEE PDK. (1996). A non-competitive ELISA for human serum insulin-like growth factor-I. *Clin. Chem.* (in press).

KRYWICKI RF AND YEE D. (1992). The insulin-like growth factor family of ligands, receptors, and binding proteins. *Breast Cancer Res. Treat.*, **22**, 7–19.

LEROITH D, BASERGA R, HELMAN L AND ROBERTS CT. (1995). Insulin-like growth factors and cancer. *Ann. Intern. Med.*, **122**, 54–59.

LEVESQUE MA, CLARK GM, YU H AND DIAMANDIS EP. (1995). Immunofluorometric analysis of p53 protein and prostate specific antigen in breast tumors and their association with other prognostic indicators. *Br. J. Cancer*, **72**, 720–727.

LONNING PE, HALL K, AAKVAAG A AND LIEN EA. (1992). Influence of tamoxifen on plasma levels of insulin-like growth factor I and insulin-like growth factor binding protein I in breast cancer patients. *Cancer Res.*, **52**, 4719–4723.

MCGUIRE WL AND CLARK GM. (1992). Prognostic factors and treatment decisions in axillary-node-negative breast cancer. *N. Engl. J. Med.*, **326**, 1756–1761.

MCGUIRE WL, TANDON AK, ALLRED DC, CHAMNESS GC AND CLARK GM. (1990). How to use prognostic factors in axillary-node-negative breast cancer patients. *J. Natl Cancer Inst.*, **82**, 1006–1015.

MCGUIRE WL, JACKSON JG, FIGUEROA JA, SHIMASAKI S, POWELL DR AND YEE D. (1992). Regulation of insulin-like growth factor-binding protein (IGFBP) expression by breast cancer cells: use of IGFBP-1 as an inhibitor of insulin-like growth factor action. *J. Natl Cancer Inst.*, **84**, 1336–1341.

MANNI A, BADGER B, WEI L, ZAENGLEIN A, GROVE R, KHIN S, HEITJAN D, SHIMASAKI S AND LING N. (1994). Hormonal regulation of insulin-like growth factor II and insulin-like growth factor binding protein expression by breast cancer cells *in vivo*: evidence for stromal epithelial interactions. *Cancer Res.*, **54**, 2934–2942.

OH Y, MULLER HL, LAMSON G AND ROSENFELD RG. (1993). Insulin-like growth factor (IGF)-independent action of IGF-binding protein-3 in Hs578T human breast cancer cells. *J. Biol. Chem.*, **268**, 14964–14971.

OWENS PC, GILL PG, DE YOUNG NJ, WEGER MA, KNOWLES SE AND MOYSE KJ. (1993). Estrogen and progesterone regulate secretion of insulin-like growth factor binding proteins by human breast cancer cells. *Biochem. Biophys. Res. Commun.*, **193**, 467–473.

PAIK S. (1992). Expression of IGF-1 and IGF-II mRNA in breast tissue. *Breast Cancer Res. Treat.*, **22**, 31–38.

PEKONEN F, NYMAN T, ILVESMAKI V AND PARTANEN S. (1992). Insulin-like growth factor binding proteins in human breast cancer tissue. *Cancer Res.*, **52**, 5204–5207.

POLLAK M, CONSTANTINO J, POLYCHRONAKOS C, BLAUER SA, GUYDA H, REDMOND C, FISHER B AND MARGOLESE R. (1990). Effect of tamoxifen on serum insulin-like growth factor I levels in stage I breast cancer patients. *J. Natl Cancer Inst.*, **82**, 1693–1697.

POLLAK MN, HUYNH HT AND LEFEBVRE SP. (1992). Tamoxifen reduces serum insulin-like growth factor I (IGF-I). *Breast Cancer Res. Treat.*, **22**, 91–100.

PRATT SE AND POLLAK MN. (1993). Estrogen and antiestrogen modulation of MCF7 human breast cancer cell proliferation is associated with specific alterations in accumulation of insulin-like growth factor-binding proteins in conditioned media. *Cancer Res.*, **53**, 5193–5198.

PRATT SE AND POLLAK MN. (1994). Insulin-like growth factor binding protein 3 (IGF-BP3) inhibits estrogen-stimulated breast cancer cell proliferation. *Biochem. Biophys. Res. Commun.*, **198**, 292–297.

SHEIKH MS, SHAO ZM, CLEMMONS DR, LEROITH D, ROBERTS CT AND FONTANA JA. (1992). Identification of the insulin-like growth factor binding proteins 5 and 6 (IGFBP-5 and 6) in human breast cancer cells. *Biochem. Biophys. Res. Commun.*, **183**, 1003–1010.

TANDON AK, CLARK GM, CHAMNESS GC, ULLRICH A AND MCGUIRE WL. (1989). HER-2/neu oncogene protein and prognosis in breast cancer. *J. Clin. Oncol.*, **7**, 1120–1128.

VALENTINIS B, BHALA A, DEANGELIS T, BASERGA R AND COHEN P. (1995). The human insulin-like growth factor (IGF) binding protein-3 inhibits the growth of fibroblasts with a targeted disruption of the IGF-I receptor gene. *Mol. Endocrinol.*, **9**, 361–367.

WINSTON R, KAO PC AND KIANG DT. (1994). Regulation of insulin-like growth factors by antiestrogen. *Breast Cancer Res. Treat.*, **31**, 107–115.

YEE D, FAVONI RE, LIPPMAN ME AND POWELL DR. (1991). Identification of insulin-like growth factor binding proteins in breast cancer cells. *Breast Cancer Res. Treat.*, **18**, 3–10.

YEE D, SHARMA J AND HILSENBECK SG. (1994). Prognostic significance of insulin-like growth factor-binding protein expression in axillary lymph node-negative breast cancer. *J. Natl Cancer Inst.*, **86**, 1785–1789.

YOUNG CY, MONTGOMERY BT, ANDREWS PE, QUI SD, BILHARTZ DL AND TINDALL DJ. (1991). Hormonal regulation of prostate-specific antigen messenger RNA in human prostatic adenocarcinoma cell line LNCaP. *Cancer Res.*, **51**, 3748–3752.

YU H, DIAMANDIS EP, ZARGHAMI N AND GRASS L. (1994). Induction of prostate specific antigen production by steroid and tamoxifen in breast cancer cell lines. *Breast Cancer Res. Treat.*, **32**, 291–300.

YU H, GIAI M, DIAMANDIS EP, KATSAROS D, SUTHERLAND DJA, LEVESQUE MA, ROAGNA R, PONZONE R AND SISMONDI P. (1995). Prostate-specific antigen is a new favourable prognostic indicator for women with breast cancer. *Cancer Res.*, **55**, 2104–2110.


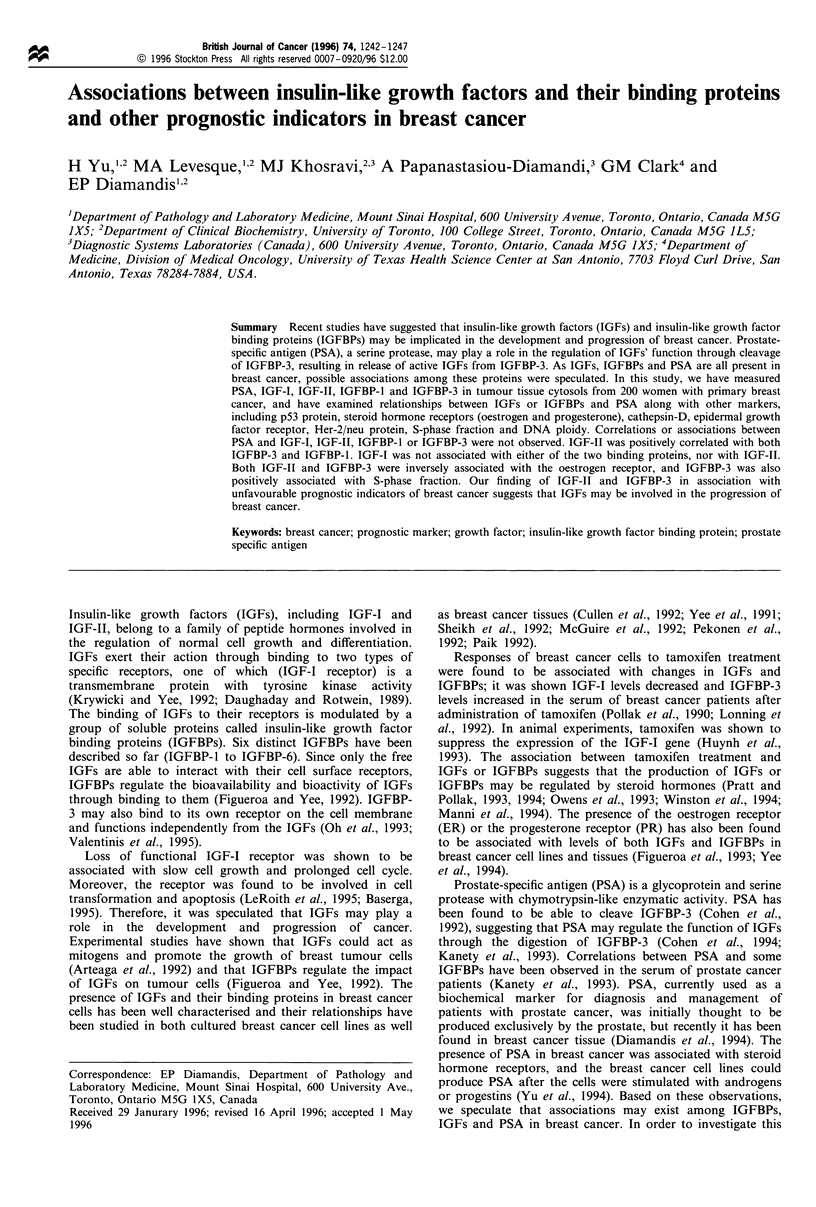

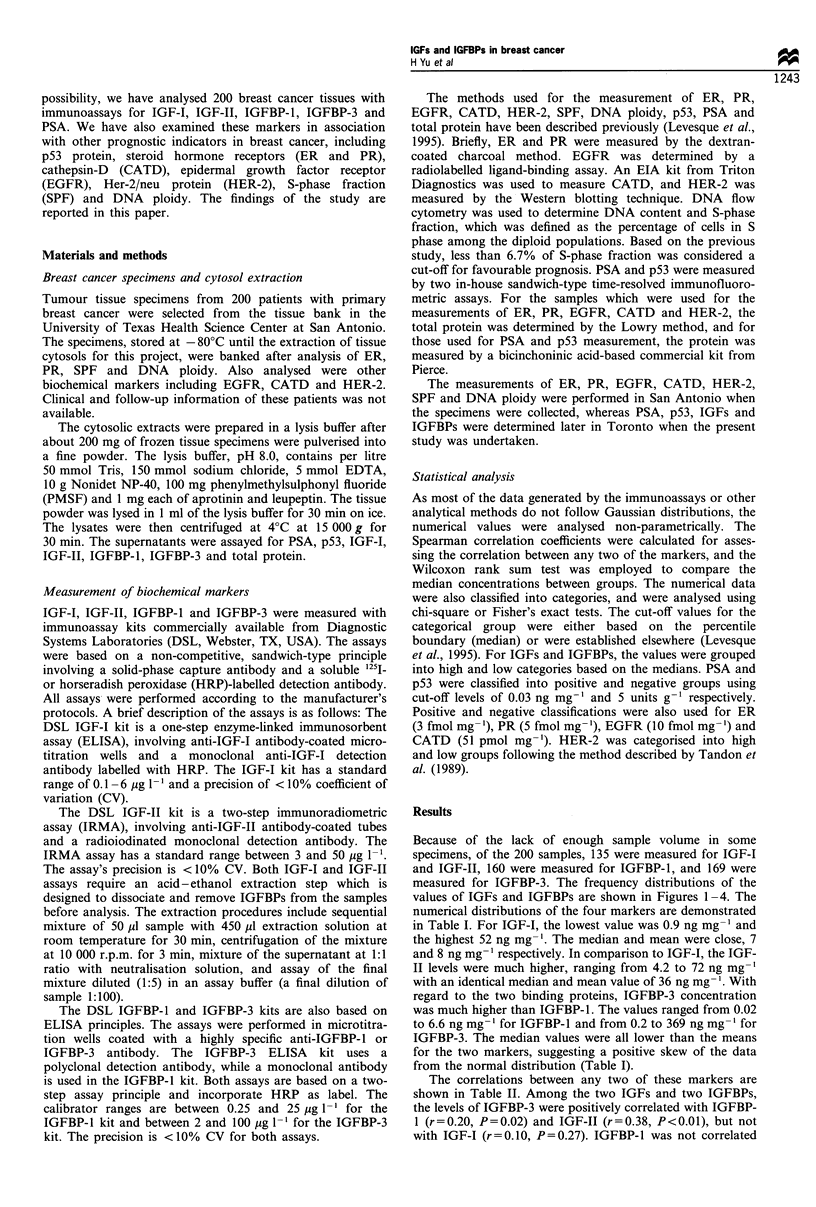

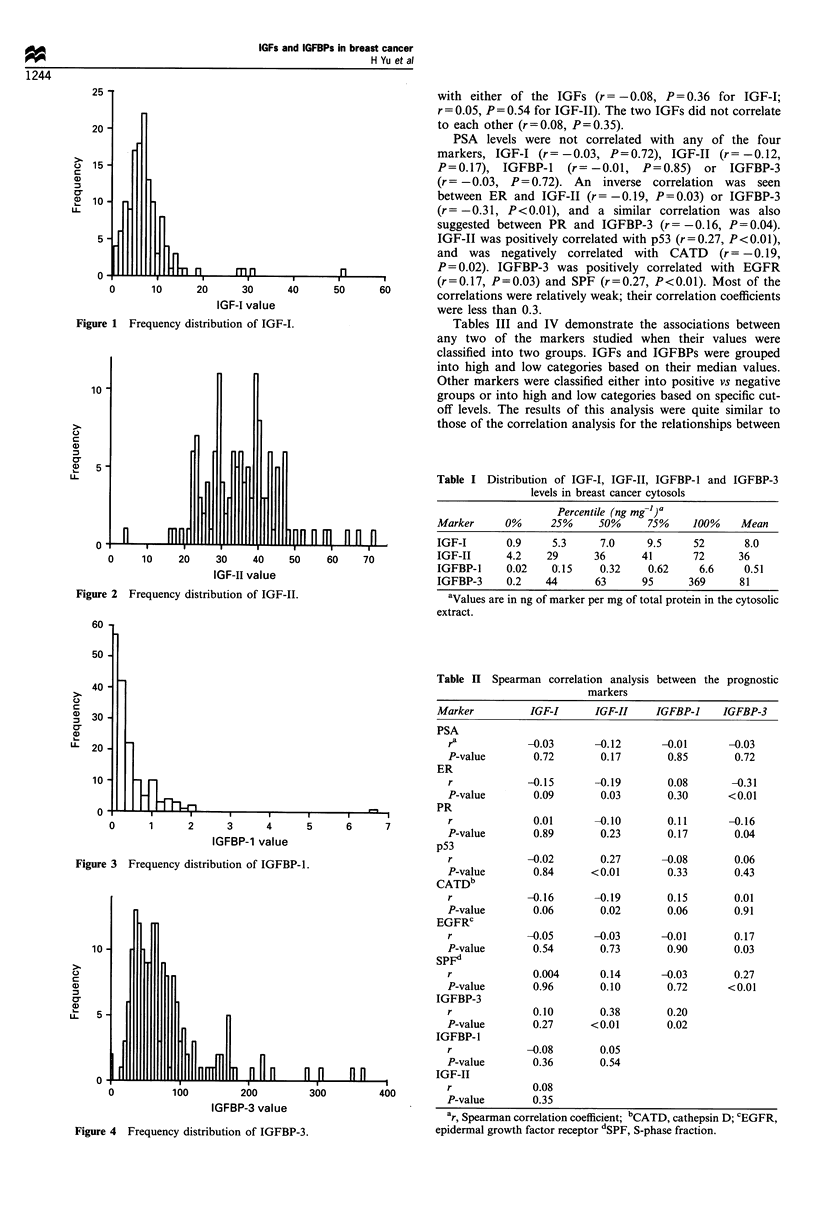

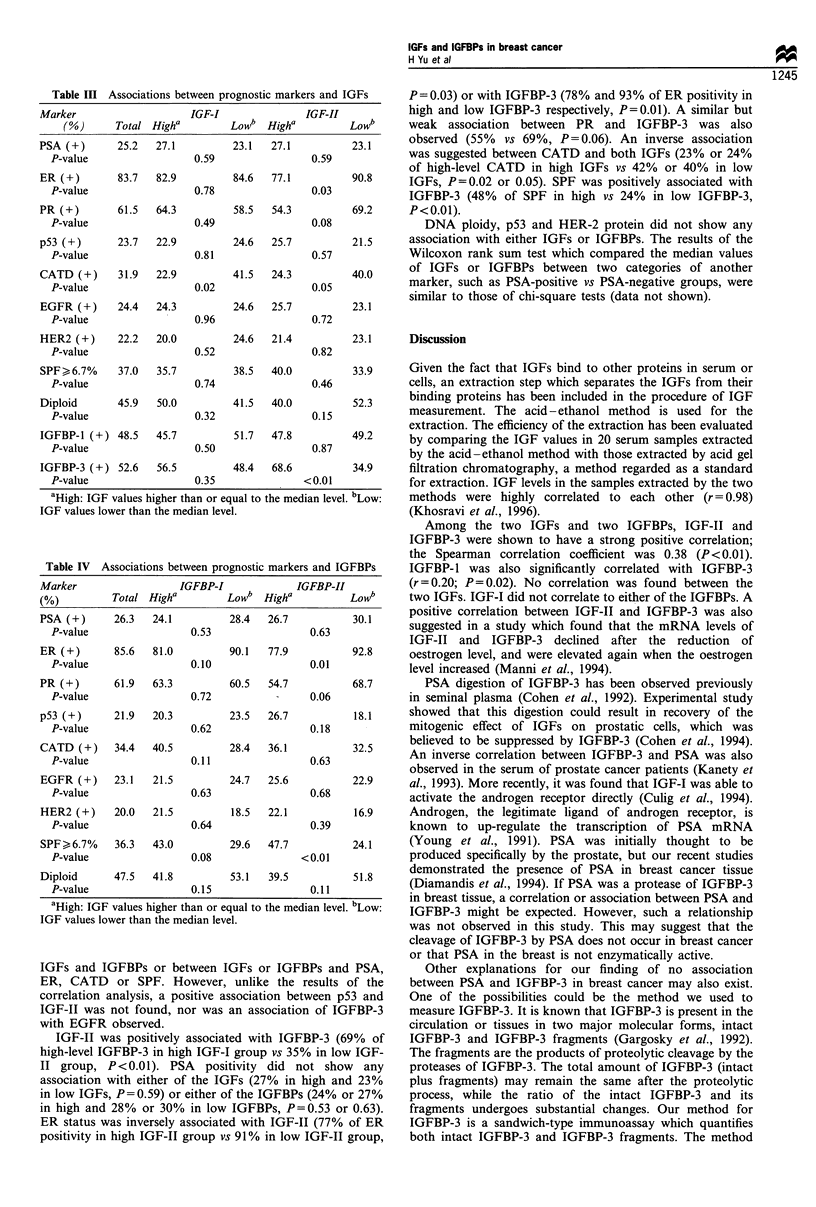

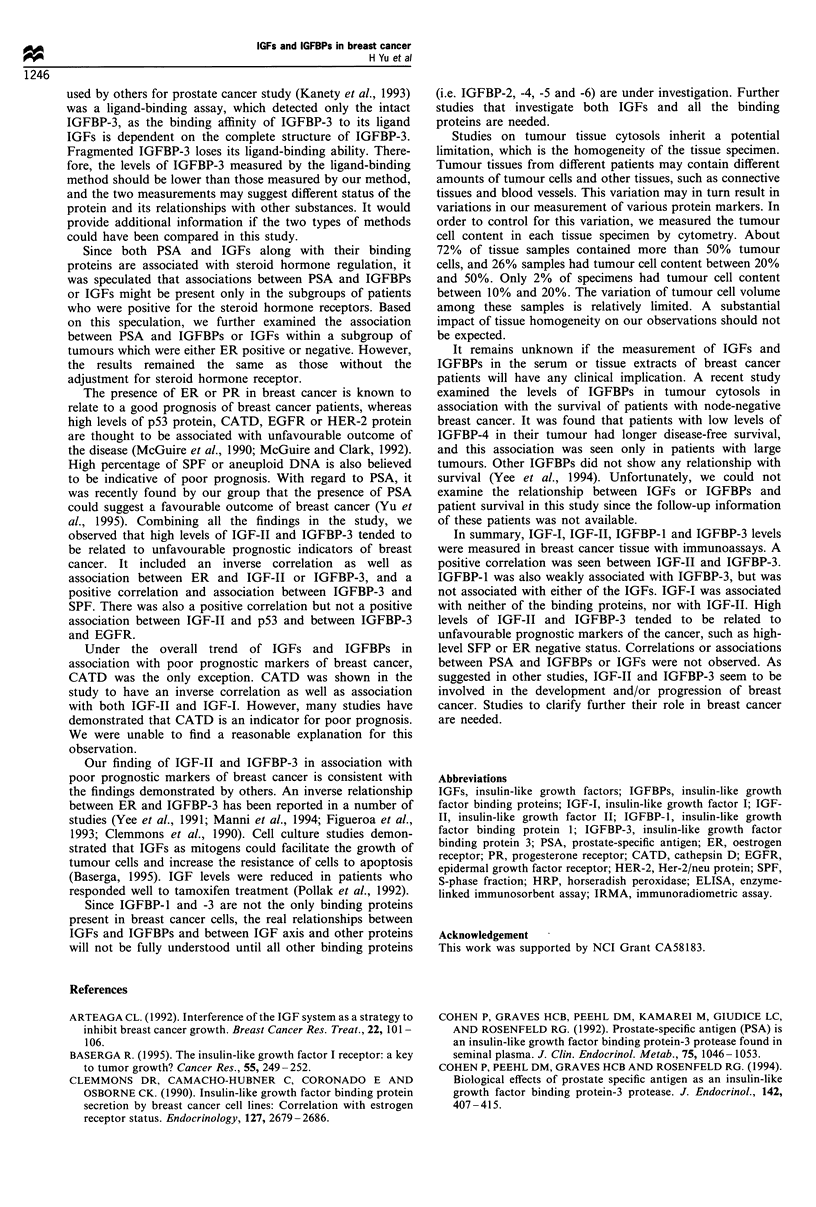

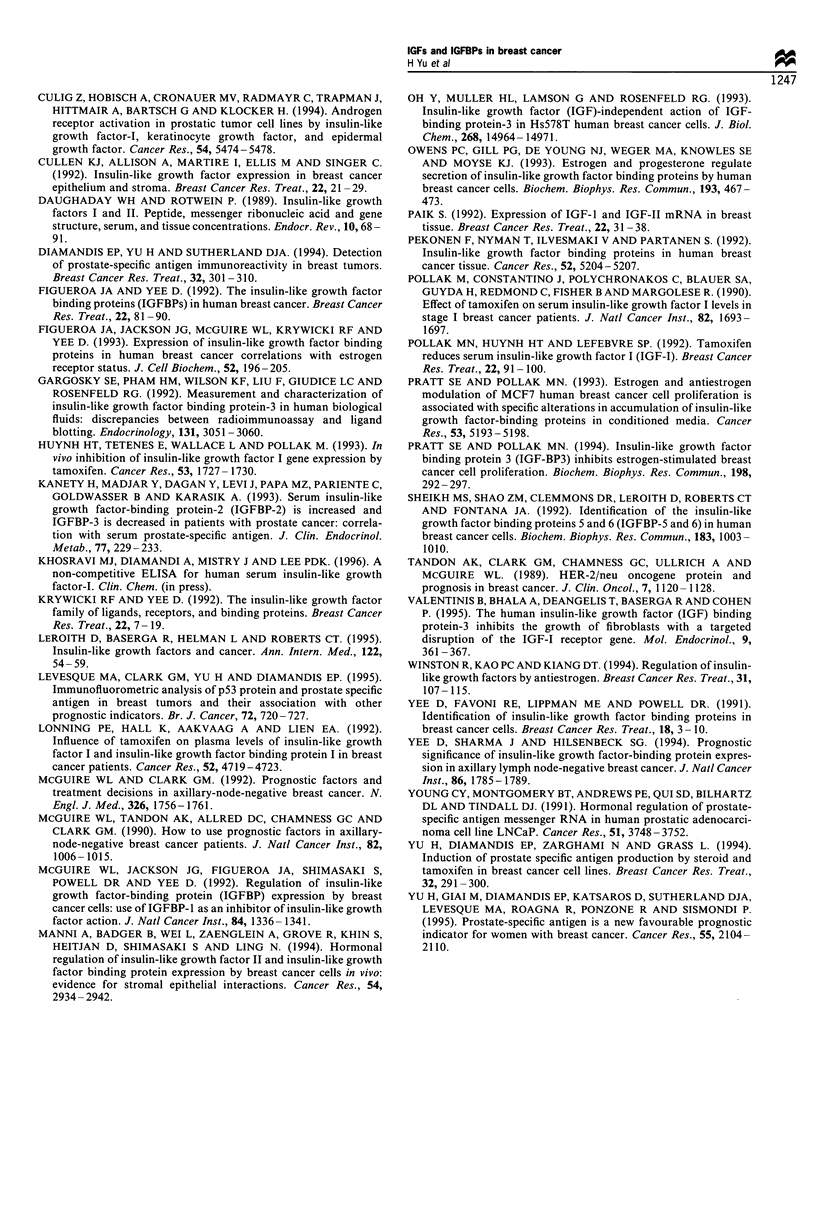


## References

[OCR_00786] Arteaga C. L. (1992). Interference of the IGF system as a strategy to inhibit breast cancer growth.. Breast Cancer Res Treat.

[OCR_00789] Baserga R. (1995). The insulin-like growth factor I receptor: a key to tumor growth?. Cancer Res.

[OCR_00795] Clemmons D. R., Camacho-Hubner C., Coronado E., Osborne C. K. (1990). Insulin-like growth factor binding protein secretion by breast carcinoma cell lines: correlation with estrogen receptor status.. Endocrinology.

[OCR_00799] Cohen P., Graves H. C., Peehl D. M., Kamarei M., Giudice L. C., Rosenfeld R. G. (1992). Prostate-specific antigen (PSA) is an insulin-like growth factor binding protein-3 protease found in seminal plasma.. J Clin Endocrinol Metab.

[OCR_00811] Cohen P., Peehl D. M., Graves H. C., Rosenfeld R. G. (1994). Biological effects of prostate specific antigen as an insulin-like growth factor binding protein-3 protease.. J Endocrinol.

[OCR_00819] Culig Z., Hobisch A., Cronauer M. V., Radmayr C., Trapman J., Hittmair A., Bartsch G., Klocker H. (1994). Androgen receptor activation in prostatic tumor cell lines by insulin-like growth factor-I, keratinocyte growth factor, and epidermal growth factor.. Cancer Res.

[OCR_00824] Cullen K. J., Allison A., Martire I., Ellis M., Singer C. (1992). Insulin-like growth factor expression in breast cancer epithelium and stroma.. Breast Cancer Res Treat.

[OCR_00829] Daughaday W. H., Rotwein P. (1989). Insulin-like growth factors I and II. Peptide, messenger ribonucleic acid and gene structures, serum, and tissue concentrations.. Endocr Rev.

[OCR_00835] Diamandis E. P., Yu H., Sutherland D. J. (1994). Detection of prostate-specific antigen immunoreactivity in breast tumors.. Breast Cancer Res Treat.

[OCR_00847] Figueroa J. A., Jackson J. G., McGuire W. L., Krywicki R. F., Yee D. (1993). Expression of insulin-like growth factor binding proteins in human breast cancer correlates with estrogen receptor status.. J Cell Biochem.

[OCR_00842] Figueroa J. A., Yee D. (1992). The insulin-like growth factor binding proteins (IGFBPs) in human breast cancer.. Breast Cancer Res Treat.

[OCR_00854] Gargosky S. E., Pham H. M., Wilson K. F., Liu F., Giudice L. C., Rosenfeld R. G. (1992). Measurement and characterization of insulin-like growth factor binding protein-3 in human biological fluids: discrepancies between radioimmunoassay and ligand blotting.. Endocrinology.

[OCR_00860] Huynh H. T., Tetenes E., Wallace L., Pollak M. (1993). In vivo inhibition of insulin-like growth factor I gene expression by tamoxifen.. Cancer Res.

[OCR_00865] Kanety H., Madjar Y., Dagan Y., Levi J., Papa M. Z., Pariente C., Goldwasser B., Karasik A. (1993). Serum insulin-like growth factor-binding protein-2 (IGFBP-2) is increased and IGFBP-3 is decreased in patients with prostate cancer: correlation with serum prostate-specific antigen.. J Clin Endocrinol Metab.

[OCR_00876] Krywicki R. F., Yee D. (1992). The insulin-like growth factor family of ligands, receptors, and binding proteins.. Breast Cancer Res Treat.

[OCR_00883] LeRoith D., Baserga R., Helman L., Roberts C. T. (1995). Insulin-like growth factors and cancer.. Ann Intern Med.

[OCR_00888] Levesque M. A., Clark G. M., Yu H., Diamandis E. P. (1995). Immunofluorometric analysis of p53 protein and prostate-specific antigen in breast tumours and their association with other prognostic indicators.. Br J Cancer.

[OCR_00894] Lønning P. E., Hall K., Aakvaag A., Lien E. A. (1992). Influence of tamoxifen on plasma levels of insulin-like growth factor I and insulin-like growth factor binding protein I in breast cancer patients.. Cancer Res.

[OCR_00916] Manni A., Badger B., Wei L., Zaenglein A., Grove R., Khin S., Heitjan D., Shimasaki S., Ling N. (1994). Hormonal regulation of insulin-like growth factor II and insulin-like growth factor binding protein expression by breast cancer cells in vivo: evidence for stromal epithelial interactions.. Cancer Res.

[OCR_00900] McGuire W. L., Clark G. M. (1992). Prognostic factors and treatment decisions in axillary-node-negative breast cancer.. N Engl J Med.

[OCR_00912] McGuire W. L., Jackson J. G., Figueroa J. A., Shimasaki S., Powell D. R., Yee D. (1992). Regulation of insulin-like growth factor-binding protein (IGFBP) expression by breast cancer cells: use of IGFBP-1 as an inhibitor of insulin-like growth factor action.. J Natl Cancer Inst.

[OCR_00906] McGuire W. L., Tandon A. K., Allred D. C., Chamness G. C., Clark G. M. (1990). How to use prognostic factors in axillary node-negative breast cancer patients.. J Natl Cancer Inst.

[OCR_00926] Oh Y., Müller H. L., Lamson G., Rosenfeld R. G. (1993). Insulin-like growth factor (IGF)-independent action of IGF-binding protein-3 in Hs578T human breast cancer cells. Cell surface binding and growth inhibition.. J Biol Chem.

[OCR_00930] Owens P. C., Gill P. G., De Young N. J., Weger M. A., Knowles S. E., Moyse K. J. (1993). Estrogen and progesterone regulate secretion of insulin-like growth factor binding proteins by human breast cancer cells.. Biochem Biophys Res Commun.

[OCR_00937] Paik S. (1992). Expression of IGF-I and IGF-II mRNA in breast tissue.. Breast Cancer Res Treat.

[OCR_00943] Pekonen F., Nyman T., Ilvesmäki V., Partanen S. (1992). Insulin-like growth factor binding proteins in human breast cancer tissue.. Cancer Res.

[OCR_00955] Pollak M. N., Huynh H. T., Lefebvre S. P. (1992). Tamoxifen reduces serum insulin-like growth factor I (IGF-I).. Breast Cancer Res Treat.

[OCR_00949] Pollak M., Costantino J., Polychronakos C., Blauer S. A., Guyda H., Redmond C., Fisher B., Margolese R. (1990). Effect of tamoxifen on serum insulinlike growth factor I levels in stage I breast cancer patients.. J Natl Cancer Inst.

[OCR_00960] Pratt S. E., Pollak M. N. (1993). Estrogen and antiestrogen modulation of MCF7 human breast cancer cell proliferation is associated with specific alterations in accumulation of insulin-like growth factor-binding proteins in conditioned media.. Cancer Res.

[OCR_00967] Pratt S. E., Pollak M. N. (1994). Insulin-like growth factor binding protein 3 (IGF-BP3) inhibits estrogen-stimulated breast cancer cell proliferation.. Biochem Biophys Res Commun.

[OCR_00974] Sheikh M. S., Shao Z. M., Clemmons D. R., LeRoith D., Roberts C. T., Fontana J. A. (1992). Identification of the insulin-like growth factor binding proteins 5 and 6 (IGFBP-5 and 6) in human breast cancer cells.. Biochem Biophys Res Commun.

[OCR_00981] Tandon A. K., Clark G. M., Chamness G. C., Ullrich A., McGuire W. L. (1989). HER-2/neu oncogene protein and prognosis in breast cancer.. J Clin Oncol.

[OCR_00985] Valentinis B., Bhala A., DeAngelis T., Baserga R., Cohen P. (1995). The human insulin-like growth factor (IGF) binding protein-3 inhibits the growth of fibroblasts with a targeted disruption of the IGF-I receptor gene.. Mol Endocrinol.

[OCR_00990] Winston R., Kao P. C., Kiang D. T. (1994). Regulation of insulin-like growth factors by antiestrogen.. Breast Cancer Res Treat.

[OCR_00997] Yee D., Favoni R. E., Lippman M. E., Powell D. R. (1991). Identification of insulin-like growth factor binding proteins in breast cancer cells.. Breast Cancer Res Treat.

[OCR_01002] Yee D., Sharma J., Hilsenbeck S. G. (1994). Prognostic significance of insulin-like growth factor-binding protein expression in axillary lymph node-negative breast cancer.. J Natl Cancer Inst.

[OCR_01009] Young C. Y., Montgomery B. T., Andrews P. E., Qui S. D., Bilhartz D. L., Tindall D. J. (1991). Hormonal regulation of prostate-specific antigen messenger RNA in human prostatic adenocarcinoma cell line LNCaP.. Cancer Res.

[OCR_01012] Yu H., Diamandis E. P., Zarghami N., Grass L. (1994). Induction of prostate specific antigen production by steroids and tamoxifen in breast cancer cell lines.. Breast Cancer Res Treat.

[OCR_01018] Yu H., Giai M., Diamandis E. P., Katsaros D., Sutherland D. J., Levesque M. A., Roagna R., Ponzone R., Sismondi P. (1995). Prostate-specific antigen is a new favorable prognostic indicator for women with breast cancer.. Cancer Res.

